# Antioxidant, Pro-Survival and Pro-Regenerative Effects of Conditioned Medium from Wharton’s Jelly Mesenchymal Stem Cells on Developing Zebrafish Embryos

**DOI:** 10.3390/ijms241713191

**Published:** 2023-08-25

**Authors:** Chiara Reina, Clara Cardella, Margot Lo Pinto, Gaia Pucci, Santina Acuto, Aurelio Maggio, Vincenzo Cavalieri

**Affiliations:** 1 Department of Biological, Chemical and Pharmaceutical Sciences and Technologies (STeBiCeF), University of Palermo, Viale Delle Scienze Ed. 16, 90128 Palermo, Italy; 2 Campus of Haematology Franco e Piera Cutino, Villa Sofia-Cervello Hospital, 90146 Palermo, Italy; 3 Zebrafish Laboratory, Advanced Technologies Network (ATeN) Center, University of Palermo, 90128 Palermo, Italy

**Keywords:** conditioned medium, Wharton’s jelly mesenchymal stem cells, zebrafish, antioxidant, regeneration, histone post-translational modifications, *nrf2*, *catalase*, *ldha*, *bcl2*

## Abstract

Conditioned media harvested from stem cell culturing have the potential to be innovative therapeutic tools against various diseases, due to their high content of growth, trophic and protective factors. The evaluation in vivo of the effects and biosafety of these products is essential, and zebrafish provides an ideal platform for high-throughput toxicological analysis, concurrently allowing the minimization of the use of mammalian models without losing reliability. In this study, we assessed the biological effects elicited by the exposure of zebrafish embryos to a conditioned medium derived from Wharton’s jelly mesenchymal stem cells. By a multiparametric investigation combining molecular, embryological, behavioural and in vivo imaging techniques, we found that exposure to a conditioned medium at a non-toxic/non-lethal dosage triggers antioxidant, anti-apoptotic and pro-regenerative effects, by upregulation of a set of genes involved in antioxidant defence (*nrf2*, *brg1*, *sirt1*, *sirt6*, *foxO3a*, *sod2* and *cat*), glycolysis (*ldha*) and cell survival (*bcl2l1*, *mcl1a* and *bim*), coupled to downregulation of pro-apoptotic markers (*baxa*, *caspase-3a* and *caspase-8*). To our knowledge, this is the first study comprehensively addressing the effects of a conditioned medium on a whole organism from a developmental, molecular and behavioural perspective, and we are fairly confident that it will pave the way for future therapeutic application.

## 1. Introduction

Mesenchymal stem cells have been used for multiple clinical applications in the field of regenerative medicine and numerous clinical trials are still in progress to provide treatment for many disparate clinical conditions, spanning from heart disease to COVID-19, among others [1,2,3,4]. On the other hand, it is commonly accepted that the benefits of these treatments primarily rely upon the broad spectrum of bioactive molecules that mesenchymal stem cells secrete into the cell growth medium, which is referred to as the conditioned medium (henceforth CM) [5,6,7,8,9]. Compared with a cell-based therapeutic treatment, a cell-free approach based on CM is arousing tremendous interest, substantially because it overcomes the adverse events commonly associated with mesenchymal stem cell administration, including rejection and malignant transformation, thereby significantly improving the patient safety profile [10]. In addition, it is noteworthy that huge amounts of CM to be used in cell-free therapies can be obtained, handled and maintained by working with a definitely smaller number of mesenchymal stem cells, compared to those usually required for cell-based therapeutic protocols [10,11]. Not to mention that CM used during the expansion of mesenchymal stem cells, otherwise discarded as a spent culture medium, can be retrieved and used as a cheaper and excellent source of bioactive factors for therapeutic purposes.

Given this premise, the preclinical evaluation in vivo of the biosafety and efficacy of these products is of pivotal importance. In this scenario, the small freshwater cyprinid *Danio rerio* commonly known as zebrafish provides an ideal platform for high-throughput toxicological analysis by allowing, at the same time, the minimization of the use of mammalian models without losing reliability. The main benefits of this vertebrate organism include ease of husbandry and maintenance under laboratory conditions, high fecundity, external fertilization, short life cycle and generation time, as well as extensive genome conservation with mammals [12,13,14,15]. Indeed, it is estimated that about 99% of embryonic-essential zebrafish genes are homologs in human embryonic development [16]. No less important, zebrafish embryos are optically transparent and relatively permeable to water-soluble molecules, facilitating non-invasive live imaging of morphogenetic processes and phenotypes following exposure to CM.

In this study, we explored the biological effects elicited by exposure of developing zebrafish embryos to CM preparations derived from Wharton’s jelly mesenchymal stem cells extracted from the human umbilical cord. By many measures, these cells display uniquely superior production of growth, trophic and protective factors compared with mesenchymal stem cells from other sources [17]. Importantly, the use of the umbilical cord for scientific research is devoid of ethical controversies, as it is usually thrown away after birth [18].

By combining molecular, cellular, embryological, behavioural and in vivo imaging techniques we appraised that CM exposure comprehensively triggers multiple favourable biological outcomes, including antioxidant, pro-survival and pro-regenerative effects, impinging on specific marker gene expression. In particular, we observed overexpression of a set of genes involved in antioxidant defence, including *nrf2*, *brg1*, *sirt1*, *sirt6*, *foxO3a*, *sod2* and *cat*, as well as the glycolytic *ldha* gene. In addition, the upregulation of pro-survival members of the *bcl2* family (*bcl2l1*, *mcl1a* and *bim*), coupled with downregulation of well-known pro-apoptotic markers (*baxa*, *caspase-3a* and *caspase-8*) was evoked by CM treatment.

## 2. Results

### 2.1. Morphological and Behavioural Effects of CM Exposure

**Figure 1:**
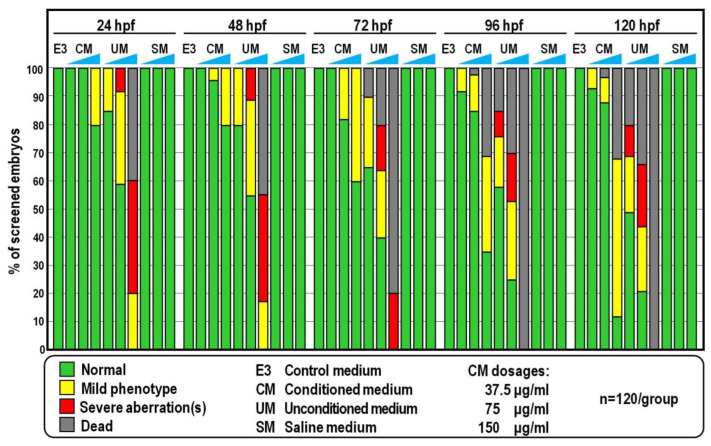
Scoring of phenotypic effects after developmental exposure of zebrafish from 6 to 120 hpf to CM, UM and SM in E3 medium, at the indicated dosages. Coloured bars in the histogram show the percentages of the observed phenotypes.

**Figure 2:**
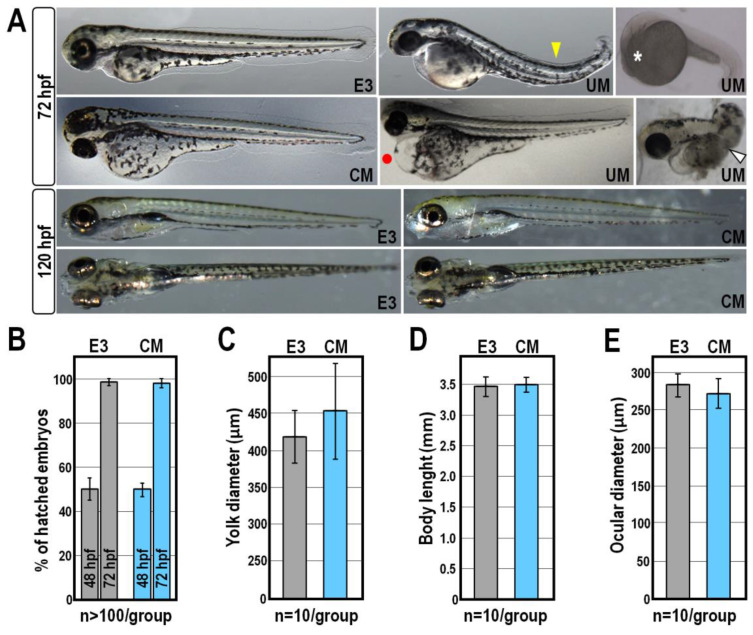
Developmental effects of zebrafish exposed to CM at 75 µg/mL. (**A**) Representative images of zebrafish embryos and larvae cultured in the absence or in the presence of equivalent amounts of CM or UM and observed at the indicated stages. The lower right corner of each image is labelled with its experimental condition. Yellow and white arrowheads indicate spinal curvature and impairment in trunk development, respectively; the asterisk indicates an underdeveloped head, while red dot indicates pericardial oedema. The two individuals shown on the bottom panels are oriented in a dorsal view, while all the others are in a lateral view; (**B**) Hatching rate of control and CM-treated zebrafish embryos at 48 and 72 hpf. Hatching rate is given as mean percentages of hatched embryos ± SD from three independent experiments; (**C**–**E**) Morphometric measurements of yolk diameter, body length and ocular diameter performed on control and CM-treated larvae at 72 hpf. Grey columns in the bar charts indicate control E3 groups, while blue columns indicate CM-exposed groups.

As a first approach to reveal the non-toxic/non-lethal biological effects induced by CM in zebrafish, developing fish embryos were exposed continuously from 6 to 120 h post-fertilization (hpf) to CM at a dosage ranging from 5 to 350 µg/mL, referred to the total protein content of CM preparations. From this analysis, we found that concentrations below 75 µg/mL did not alter zebrafish embryogenesis (Figure 1 and Figure 2). Indeed, the vast majority of embryos exposed to CM at this dosage exhibited a normal phenotype as well as survival and hatching rates broadly similar to those of control groups of fish reared in standard E3 medium (Figure 1 and Figure 2; *p* > 0.25). Compared to control groups at 72 hpf, roughly 18% (n = 22/120) of CM-treated embryos showed bigger yolks (Figure 1 and Figure 2A). However, measurement of yolk diameter showed that this difference was statistically irrelevant (Figure 2C; *p* > 0.15) and defects in yolk reabsorption were mostly recovered at 120 hpf, when more than 88% (n = 106/120) of CM-treated larvae did not show any morphological alteration nor overt differences compared to control fish at the same stage reared in E3 (Figure 1 and Figure 2A). Moreover, additional morphometric measurements performed on control and CM-treated embryos at 72 hpf highlighted that the body length and ocular diameter were largely unaffected by CM exposure (Figure 2D,E; *p* > 0.65 and *p* > 0.15, respectively). At the higher dose of 150 µg/mL, significant fractions (about 20% to 50%) of CM-treated embryos displayed durable developmental delay and more than 30% of them (n = 39/120) died at 120 hpf (Figure 1).

Overall, this experiment was repeated using distinct CM preparations derived from four randomly selected donors, eliciting broadly similar results (not shown). Much to our surprise, however, a significant fraction of embryos exposed to equivalent amounts of unconditioned medium (UM) did not attain developmental milestones and displayed a variety of sublethal and severe deformities including spinal curvature, pericardial oedema and missing head or tail, coupled to a mortality rate ranging from 20% to 100% at 120 hpf (Figure 1 and Figure 2A). These failures were not due to the ionic strength conferred by UM, since embryos were all viable and phenotypically normal when exposed to equal amounts of SM, a solution having the same inorganic salt composition and concentration as UM (Figure 1).

**Figure 3:**
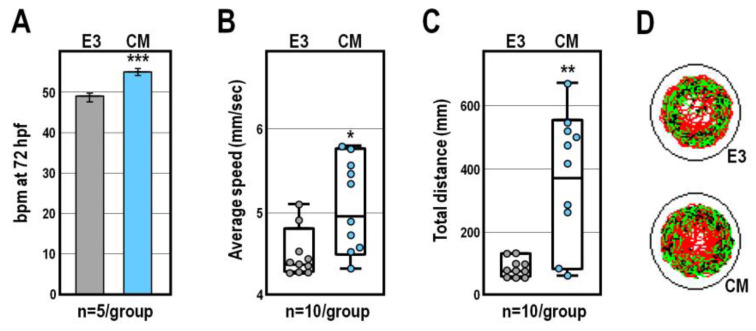
Analysis of heartbeat and locomotor activity following exposure to CM at 75 µg/mL. (**A**) Heartbeat measurement represented as average values of beats per minute (bpm) in control and CM-treated zebrafish larvae at 72 hpf; (**B**,**C**) Analysis of the average swimming speed and total distance travelled of control and CM-treated embryos at 119 hpf; control groups are indicated in grey, while CM-exposed groups are indicated in blue in each graph; (**D**) Representative cumulative plots of the position, velocity and trajectory of control and CM-treated larvae during 30 min of behavioural recording. Significant differences against the E3 control group are indicated either by * (*p* < 0.05), ** (*p* < 0.01) or *** (*p* < 0.001).

CM exposure was associated with a relatively small but reproducible speeding of cardiac activity, as indicated by the increase of about 12% (*p* < 0.001) in heartbeat frequency of CM-treated larvae at 72 hpf, compared to that of control larvae at the same stage (Figure 3A). Accordingly, by using a video tracking system to measure the locomotor parameters of 119 hpf-old larvae, we found that exposure to CM evoked hyperactivity, so that both the average swim speed and total distance travelled were specifically increased (*p* < 0.05 and *p* < 0.01, respectively) compared to control larvae at the same stage (Figure 3B,C). Beyond these differences, control and CM-treated larvae exhibited similar global patterns of movement trajectory (Figure 3D).

In light of the results obtained from these analyses, we decided to perform the subsequent experiments using CM at the dosage of 75 µg/mL.

### 2.2. CM Exposure Impacts on the Antioxidant Gene Network

According to the literature, the behavioural effects we observed following CM exposure might be attributed either to the downregulation of the *brain-derived neurotrophic factor* (*bdnf*) gene [19,20,21] or to the overexpression of genes involved in redox homeostasis [22,23,24,25,26,27].

**Figure 4:**
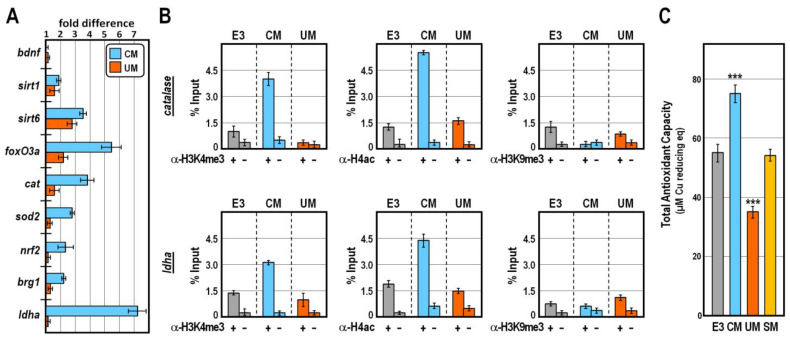
Effect of CM exposure on the redox homeostasis of zebrafish. (**A**) Changes in gene expression level of *bdnf*, *ldha* and redox marker genes assessed by qPCR in 30 hpf-old embryos exposed to CM or UM. Data are indicated as fold differences in transcript abundance with respect to control embryos, at the same stage of development, reared in E3. Error bars are standard errors for the qPCR replicates. Oligonucleotide primer pairs used for qPCR reactions and amplicon lengths are indicated in Table S1; (**B**) ChIP-qPCR analysis of the *cat* and *ldha* promoter occupancy by H3K4me3, H4ac and H3K9me3. ChIP assays were performed on chromatin extracted from control, CM- and UM-treated embryos at 30 hpf. Purified chromatin was precipitated with the indicated antisera or incubated without adding antibodies, followed by qPCR amplification of *cat* and *ldha* promoter fragments using the oligonucleotide primer pairs listed in Table S1. Data are normalized according to the percent of input method. Bars are as in (**A**); (**C**) Total antioxidant capacity expressed as μM Copper reducing equivalents in embryos at 30 hpf exposed to the indicated experimental conditions. Values are mean ± SD and significant difference against the E3 control group is indicated by *** (*p* < 0.001). In the bar charts, grey columns indicate control E3, blue columns indicate CM-exposed, red columns indicate UM-treated, and orange columns indicate SM-treated groups of fish, respectively.

To distinguish between these two possibilities, we explored by qPCR the expression of selected marker genes in control and CM-treated embryos at 30 hpf, when tissue-specific epigenetic and transcriptional programs have been established [12,28,29,30]. Strikingly, while CM-exposure did not appreciably change the *bdnf* transcript level with respect to controls (Figure 4A), we found that the mRNA abundances of several genes involved in the first line of antioxidant defence were significantly increased in CM-treated embryos (Figure 4A). This group of genes included those coding for the Nrf2 master regulator of antioxidant response [31], the chromatin remodeler Brg1 [32], the Sirt1 and Sirt6 lysine deacylases [33], the FoxO3a transcription factor [34] and the reactive oxygen species (ROS)-detoxifying enzymes superoxide dismutase-2 and catalase [35]. A similar trend, although to a lesser extent, was detected for some of these genes in UM-treated embryos (Figure 4A). In addition, we found that the expression of the glycolytic gene coding for lactate dehydrogenase-A enzyme, which converts pyruvate to lactate, was robustly and specifically increased by CM treatment (Figure 4A).

Chromatin immunoprecipitation assay confirmed that in CM-, but not UM-, treated embryos at 30 hpf the promoter of *cat* and *ldha* genes were significantly enriched in histone post-translational modifications associated with permissive chromatin, such as trimethylation of histone H3K4 and acetylation of histone H4, in the absence of the heterochromatic mark trimethylated H3K9 (Figure 4B). Such an epigenetic signature typically foreshadows high propensity for gene expression [30,36,37], thus justifying the observed increase in mRNA abundance for the mentioned genes in CM-treated embryos. In strict accordance, the total antioxidant capacity, which results from the cumulative action of all the antioxidant species, was specifically improved in CM-treated embryos at 30 hpf (Figure 4C; *p* < 0.001). By contrast, sibling embryos exposed to equivalent amounts of UM exhibited a marked reduction in their total antioxidant capacity (Figure 4C; *p* < 0.001), while no relevant changes occurred in embryos grown in SM (Figure 4C; *p* > 0.75). Altogether, these findings suggest that CM and UM exposures impinge disjointly on the antioxidant defence system, with CM exposure specifically exhibiting a favourable impact on the redox homeostasis of zebrafish.

### 2.3. CM Exposure Establishes Antioxidant Defence by Reducing ROS Level

**Figure 5:**
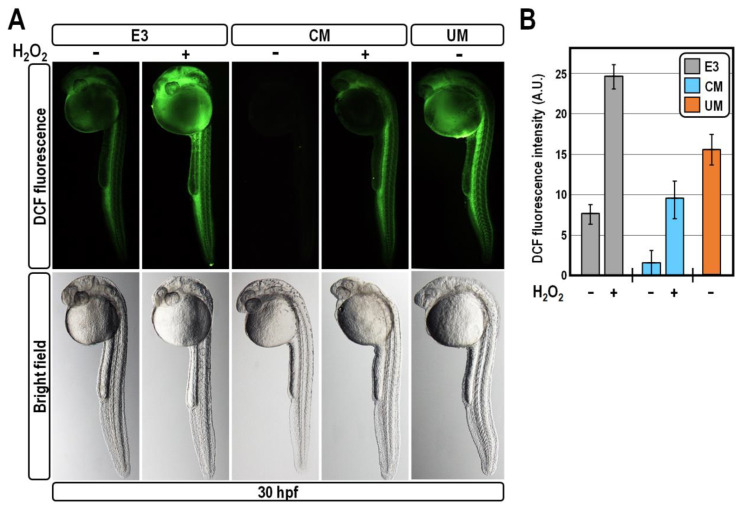
CM exposure protects against hydrogen peroxide-induced oxidative stress by reducing ROS level. (**A**) Representative images of 30 hpf-old zebrafish embryos observed following DCFH-DA staining to measure ROS level at the experimental conditions indicated on top of the figure. Images for each embryo are shown under fluorescence and bright-field optics; (**B**) Mean DCF fluorescent intensity quantified using the Image J software in the whole embryos and expressed as arbitrary units.

In principle, the observed upregulation of antioxidant gene expression established following CM exposure could be either a response to allow the efficient removal of excessive oxidant species accumulation or a protective mechanism preventing the generation of oxidative stress. We reasoned that the concomitant overexpression of *ldha* in CM-treated embryos would argue in favour of the latter condition, as it would hamper the entry of pyruvate into mitochondria for oxidative phosphorylation. This, coupled with the coordinated overexpression of the antioxidant gene battery, would in turn hinder both the production and accumulation of mitochondrial ROS. To test this hypothesis, we quantified the ROS content in fish embryos at 30 hpf, using the DCFH-DA vital staining. As anticipated, CM exposure almost nullified the ROS-related fluorescence intensity, bringing it below the baseline level of unperturbed control embryos, thus confirming a reduced susceptibility of CM-treated embryos to oxidative damage (Figure 5). In comparison, treatment with UM greatly increased the accumulation of ROS throughout the embryo (Figure 5), which is indicative of oxidative stress.

These results prompted us to appraise whether CM treatment shields fish embryos from acute oxidative injury. For this purpose, control and CM-treated embryos at 30 hpf were exposed to the pro-oxidant hydrogen peroxide at the concentration of 50 µM for 20 min. This treatment provoked a strong and expected increase of the ROS amount in control embryos, as indicated by their brighter DCF-fluorescent intensity compared to sibling untreated specimens (Figure 5). Remarkably, in CM-exposed embryos, hydrogen peroxide treatment was unable to efficiently drive the elevation of ROS level, which remained in the same order of magnitude as unperturbed controls (Figure 5). Altogether, these findings lead us to conclude that exposure of zebrafish embryos to CM specifically accounts for the establishment of a self-protective mechanism of ROS clearing.

### 2.4. CM Exposure Promotes Pro-Survival Effect in Developing Zebrafish

**Figure 6:**
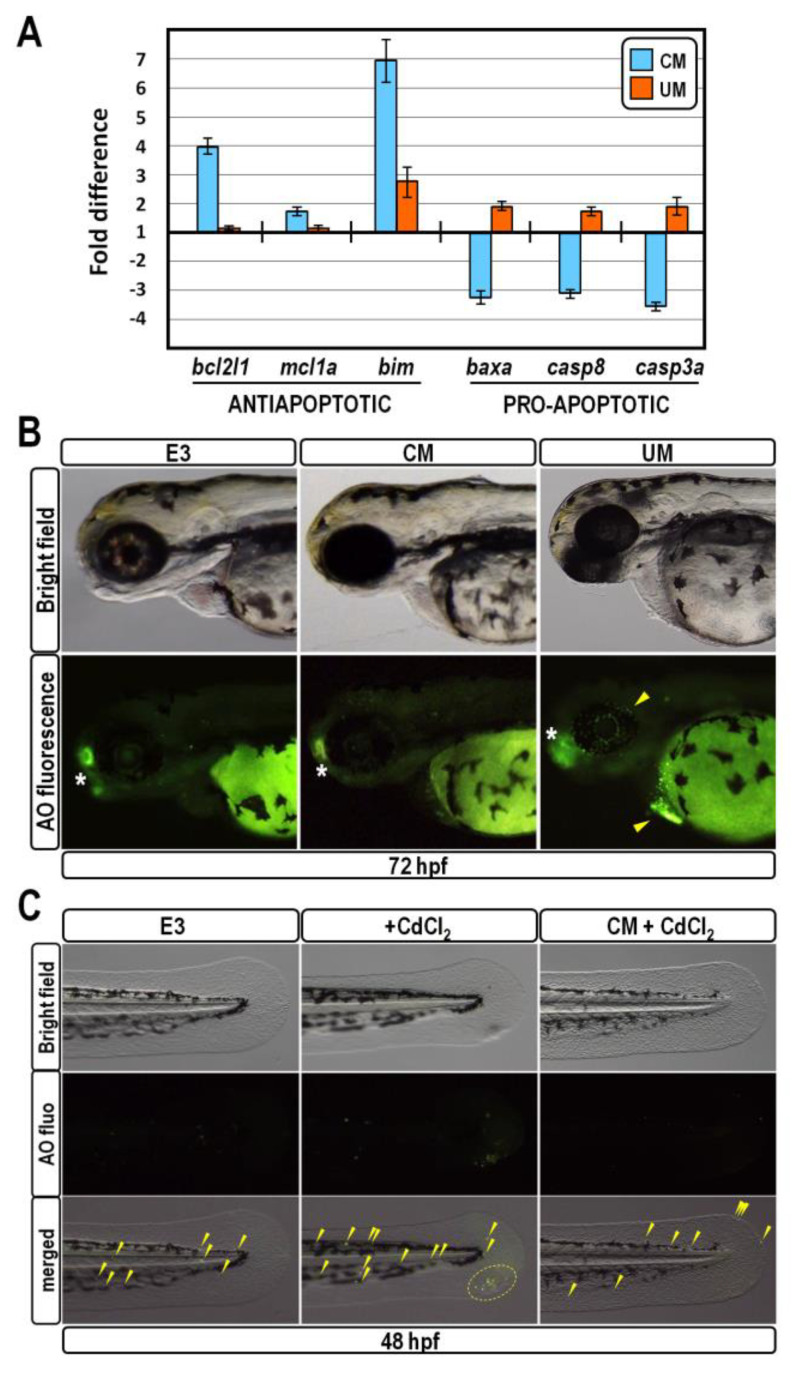
CM exposure provokes protective anti-apoptotic effect. (**A**) Changes in gene expression level of anti- and pro-apoptotic marker genes assessed by qPCR in 30 hpf-old embryos exposed to CM or UM. Data are shown as described in Figure 4A; (**B**) Analysis of territorial apoptosis in representative control, CM- and UM-treated zebrafish larvae at 72 hpf stained with acridine orange. Asterisks mark apoptosis normally occurring in olfactory placodes, while yellow arrowheads indicate aberrant apoptotic foci in ocular and pericardial regions aroused by UM treatment; (**C**) Detection of apoptosis in the posterior trunk and tail of 48 hpf-old zebrafish embryos reared in the indicated conditions. Images for each embryo are shown under bright field optics, fluorescence and aggregate merging from the top downwards. Yellow dashed circles and arrowheads indicate apoptotic foci.

Since redox homeostasis and cell death molecular pathways are strictly intermingled [38,39], we suspected that the reduction of ROS level triggered by CM exposure could provide a supportive anti-apoptotic effect. Concordantly, by means of qPCR analysis in cDNA samples derived from embryos at 30 hpf, we found that CM exposure elicited upregulation of pro-survival members of the *bcl2* family, coupled to downregulation of the well-known pro-apoptotic markers *baxa*, *caspase-3a* and *caspase-8* (Figure 6A). An almost diametrically opposite effect was observed in embryos exposed to UM, indicating an abnormally increased apoptosis rate in these embryos (Figure 6A).

Next, we used the acridine orange-fluorescent staining to determine the spatial distribution of apoptotic cells in live 72 hpf-old embryos. At this stage, the olfactory placodes are normally marked by a high apoptotic rate [40], which provides an internal control for the assay. Acridine orange staining confirmed that overexpression of pro-apoptotic markers triggered by UM-treatment did correlate with the appearance of supernumerary foci of cell death in ocular and pericardial regions (Figure 6B). In sharp contrast, CM-treated embryos did not change their spatial apoptotic pattern with respect to unperturbed controls, again suggesting a pro-survival effect (Figure 6B).

To assess whether CM treatment might protect fish embryos from acute exposure to a pro-apoptotic stressor, control and CM-treated embryos at 24 hpf were exposed to 12 µM Cadmium chloride for 24 h, and the occurrence of apoptotic cells was determined by acridine orange staining at 48 hpf. In accordance with literature reports [41], Cadmium treatment increased the number of apoptotic foci in the trunk and tail of control embryos (Figure 6C). Strikingly, in CM-exposed embryos, Cadmium treatment was unable to successfully multiply the number of apoptotic foci, which was proportionate to those of unperturbed controls (Figure 6C). Altogether, these findings strongly suggest that exposure of zebrafish embryos to CM specifically accomplishes a fully protective role against aberrant apoptosis caused by Cadmium.

### 2.5. Stimulatory Effect of CM Exposure on Caudal Fin Regeneration

**Figure 7:**
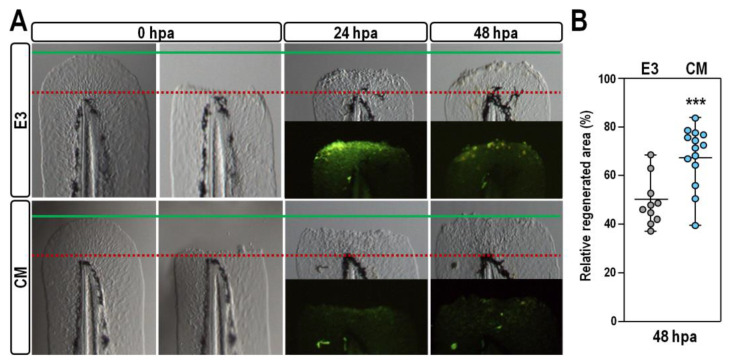
CM exposure stimulates caudal fin regeneration after amputation. (**A**) Representative images of caudal tails resected from control and CM-treated embryos at 48 hpf and allowed to regenerate for 48 hpa. For each larva, the red dashed line indicates the plane of amputation at 0 hpa, while the green line indicates the posterior edge of the caudal fin just before amputation. Fluorescence images show acridine orange staining of regenerating fins at 24 and 48 hpa; (**B**) Changes in the relative area of regenerating caudal fin in control and CM-treated larvae at 48 hpa. Values are indicated in grey for control E3 and in blue for CM-treated larvae. Significant difference against the E3 control group is indicated by *** (*p* < 0.001).

To ascertain if exposure to CM impacts the regenerative capacity of larval zebrafish, caudal fin primordia of control and CM-treated embryos at 48 hpf were resected and were allowed to regenerate for 48 h post-amputation (hpa). At this time point, compared to control un-amputated fish, at the same stage, two-thirds (n = 10/15) of control larvae have undergone fin regeneration, restoring on average ~50% of their fin area (Figure 7A,B). Strikingly, at the same time point, all but one of the CM-treated larvae (n = 14/15) significantly increased the rate of restoration of their fin both in size and shape, compared to controls (Figure 7A,B; *p* < 0.001). Moreover, as expected from the above-mentioned observation, acridine orange staining revealed a substantially reduced number of apoptotic cells in the injured fin of CM-treated fish compared with controls (Figure 7A), suggesting that probably CM exposure enhances fin regrowth by provoking an imbalance between cell death progression and cell proliferation occurring during regeneration.

## 3. Discussion

In the field of regenerative and protective medicine, cell-free approaches involving CM obtained from various sources are rapidly emerging as an attractive therapeutic option, as they override the drawbacks related to the handling and delivery of intact mesenchymal stem cells to allogeneic recipients [9,42,43,44]. In recent years, CM derived from Wharton’s jelly mesenchymal stem cells extracted from the human umbilical cord has received significant attention due to the high content of bioactive molecules making it theoretically optimal for treating various disorders and injuries [45]. However, given that most of the published studies have been performed in cell culture systems, the potential mechanisms influenced by CM components in a whole organism remained unclear or poorly explored. In sharp contrast, we assessed the biological effects occurring during exposure of developing zebrafish embryos and larvae to CM preparations derived from Wharton’s jelly mesenchymal stem cells. The first and foremost finding emerging from this analysis is that CM treatment does not seem to inflict overt toxicity or lethality to zebrafish larvae, nor does it cause gross morphological defects up to the concentration of 75 µg/mL. In fact, although we observed a slight delay in yolk reabsorption in a small fraction of CM-treated embryos at 72 hpf, this condition was mostly recovered at 120 hpf, suggesting that this minor defect is to some extent reversible. The fact that the hatching rate did not vary during CM exposure, coherently reaffirms that there was no disturbance in the anatomy and physiology of the CM-treated embryos. Indeed, the hatching process is a crucial stage during zebrafish embryogenesis, requiring coordinated expression of hatching enzymes and swinging locomotion of the developing embryos to ensure the finally successful breaking of the chorion [46,47,48].

Exposure to CM evokes hyperactivity in zebrafish larvae, as indicated by the increase in the average of heartbeat frequency and locomotor parameters with respect to control larvae. Although these findings could suggest a potential adverse outcome of CM exposure, two lines of evidence lead us to exclude that this is the case. First, morphological and morphometrical analysis highlighted the absence of developmental defects, such as muscle disorganization, in CM-treated individuals, which is consistent with the finding that behaviour is among the most sensitive endpoints in zebrafish toxicity screenings [14]. Second, we did not observe disorganization in the global swimming pattern of CM-treated larvae, thus ruling out any behavioural response associated with increased fear or anxiety [49].

Remarkably, a number of reports from other authors linked hyperactivity either to exposure to antioxidants or to upregulation of antioxidant gene expression [22,23,24,25,26,27]. In line with this evidence, the hyperactivity observed in CM-treated larvae strictly associates with a reinforced antioxidant performance at the epigenetic and transcription level, as well as in the overall value of their antioxidant capacity. However, since zebrafish larval hyperactivity can be elicited via several neurological pathways [50], the exact molecular mechanism(s) underlying the relationship between hyperactivity and antioxidant response following CM exposure needs to be further investigated.

Contrary to what the name might suggest, redox homeostasis is a dynamic process acting through a highly responsive system that senses changes in cellular redox status and realigns metabolic activities to restore redox balance, thus regulating a plethora of biological responses [51]. A key regulator of this molecular system is the Nrf2 transcription factor. Under oxidative stress, Nrf2 translocates into the nucleus, where it engages in a complex containing the Brg1 chromatin remodeler and binds to the promoter of antioxidative response genes, stimulating their transcription [31,32]. Sirtuin lysine deacylases also appeared to be involved in the activation of the same antioxidant gene battery by means of distinct mechanisms, including modulation of the FoxO family of transcription factors [33]. Altogether, these mechanisms lead to enhancement in cellular antioxidant capacity and scavenging of ROS and unsafe oxidative metabolites.

Our findings collectively indicate that CM exposure specifically accounts for the establishment of an antioxidant milieu not only through impacting favourably on the expression of genes belonging to both the mentioned regulatory circuits, but also by eliciting upregulation of *ldha* gene expression. LDHA is the direct handler of pyruvate to lactate transforming, which is associated with NAD+ regeneration [52]. This process also contributes to redox homeostasis maintenance because it directly supplies reducing power against ROS [53,54] and ensures a low demand of mitochondria to produce ATP through oxidative phosphorylation, thereby hampering the elevation of mitochondrial ROS [55,56]. Intriguing evidence from other authors showed a relation between LDHA function and cell survival, indicating that *ldha* gene expression could be intensified by exposure to antioxidant compounds [57], and that it anticorrelates with oxidative stress and cell death occurrence [58]. In fact, oxidative stress and apoptosis share an interdependent relationship, since many of the chemical and physical stimuli capable of inducing apoptosis are known to evoke or further aggravate oxidative stress [38], while antioxidant enzymes can counteract apoptotic signalling pathways leading to cell death [39]. Concordantly, our findings strongly suggest that exposure of zebrafish embryos to CM specifically plays a fully protective role against aberrant apoptosis.

Regeneration of caudal fin tissue in zebrafish is a well-orchestrated process in which the injured or lost structure is completely replaced [59]. Notably, the oxidative stress arisen by fin amputation is the most important hindrance for regeneration, since excessive ROS around the wound damages the surrounding tissues, provoking retardation of wound closure [60]. It follows that, once again, the antioxidant protection effect imposed by CM exposure could prompt fin regeneration following amputation. Similar effects have been reported in other studies describing that exposure of zebrafish larvae to antioxidant substances promotes faster fin regeneration, further corroborating our evidence [61,62]. Additionally, owing to the notion that a coordination and balance among cell proliferation and apoptosis is crucial for fin regeneration in zebrafish [63], the observed decrease in the apoptotic activity in response to CM exposure could certainly account for the apparent regrowth stimulation of the amputated fin. Future investigations will shed more light on the potential effects of CM exposure on cell proliferation during regeneration.

Our current efforts are aimed at the identification of the factors present in CM responsible for the observed effects. To this purpose, we are investigating the protein composition of the CM samples used in this study, by means of multiplex ELISA protein detection arrays. Furthermore, since CM bioactive factors can be roughly separated into two basic components, namely soluble factors and membrane-bound factors, additional experiments are currently planned to systematically determine whether soluble factors, membrane-bound factors or both are responsible for the observed effects in zebrafish. Finally, also of future interest will be the analysis of the long-term effects of the developmental exposure to CM on subsequent zebrafish lifecycle periods, from the post-larval stages to adulthood. Obviously, this specific investigation will require dedicated funds and personnel time, as well as prior ethical authorization, according to the current rules on the protection of animals used for scientific purposes.

## 4. Materials and Methods

### 4.1. Preparation of Conditioned Medium

Wharton’s jelly mesenchymal stem cells were isolated from the umbilical cords of four distinct donors with written informed consent according to the tenets of the Declaration of Helsinki and with a protocol approved by the Ethical Committee at the Azienda Ospedaliera Ospedali Riuniti Villa Sofia-Cervello (approval number 331-AOR2016). Wharton’s jelly mesenchymal stem cells at the third passage were cultured at a density of 4.000 cells/cm^2^ in Dulbecco’s Modified Eagle’s low-glucose medium (DMEM, EuroClone) supplemented with 5% human platelet lysate (EuroClone) to 80% confluence in T75 flasks. Then, the attached cells were gently washed twice with standard phosphate-buffered saline, the complete medium was replaced with basal DMEM and the resulting CM was harvested following 48 h of incubation. The collected CM was centrifuged for 10 min at 3000 rpm, 0.2 μm filtered to remove cell debris and concentrated ~50-fold by ultrafiltration using an Amicon Ultra-15 centrifugal filter device with 10 kDa nominal molecular weight limit (Millipore). Total protein quantification was performed by Bradford assay in the concentrated CM, and the mixture was aliquoted and stored at −80 °C until use.

### 4.2. Zebrafish Maintenance and Breeding

The Zebrafish Laboratory of the Advanced Technologies Network Center at the University of Palermo is allowed to use zebrafish (*Danio rerio*) for scientific research by the Italian Ministry of Health (aut. prot. no. 24/2023-UT dated 13 June 2023 that replaces the previous aut. prot. no. 06/2017-UT dated 30 March 2017) and by the Provincial Health Authority of Palermo (code no. 053PA414 dated 6 June 2019). Wild-type (AB strain) zebrafish adults were purchased from the European Zebrafish Resource Center (Karlsruhe Institute of Technology, Eggenstein-Leopoldshafen, Germany) and maintained in a recirculating aquaculture system (Tecniplast) under standard conditions, according to National (Italian D.lgs 26/2014) and European (2010/63/EU) animal welfare laws.

On the previous day of spawning, fish were placed in sloped breeding tanks (Tecniplast) with the males and females separated by a divider. On the next day, the dividers were removed and spawning was initiated after the onset of the light phase of their photoperiod. Viable and synchronously developing embryos were selected under a M80 stereomicroscope (Leica Microsystems, Milan, Italy) and kept at 28.5 ± 0.5 °C in sterile E3 medium (5 mM NaCl, 0.33 mM CaCl_2_, 0.17 mM KCl, 0.33 mM MgSO_4_) for subsequent experiments.

All the experimental protocols described in this study were carried out exclusively with zebrafish embryos and larvae up to 120 hpf. At this stage of their life cycle, zebrafish are not capable of independent feeding and therefore are not subjected to the Italian (D.lgs 26/2014) and European (2010/63/EU) rules on the protection of animals used for scientific purposes.

### 4.3. Zebrafish Embryo Exposure to CM and Evaluation of Phenotype

Developing embryos were placed in 96-well culture plates (1 embryo/well), maintained at a temperature of 28.5 ± 0.5 °C and exposed continuously from 6 to 120 hpf to CM preparations derived from four distinct umbilical cords and added to the E3 medium at dosages ranging from 5 to 350 µg/mL referred to the total protein content of CM samples. Control groups for these experiments included sibling embryos exposed to equivalent amounts of UM (which is the basal DMEM), to SM (a solution having the same inorganic salt composition and concentration as DMEM: 265 mg/L CaCl_2_ · 2 H_2_O, 0.1 mg/L Fe(NO)_3_, 97.72 mg/L MgSO_4_, 0.4 g/L KCl, 6.4 g/L NaCl) or to standard E3 medium. Twenty-four replicates were run for each experimental condition, with each replicate consisting of a single well containing a viable developing embryo and the given treatment in 200 µL of E3 medium. Each experiment was repeated five times using independent batches of embryos. Treated and control embryos were carefully examined by daily observation under a M205-FA multidimensional stereomicroscope (Leica Microsystems, Milan, Italy) until 120 hpf, to estimate the mortality rate as well as developmental delay and phenotypic alterations. Digital images were captured and the ImageJ software (https://imagej.nih.gov/ij/ (accessed on 3 March 2023)) was used to analyse morphometric and growth parameters, including larval body length, ocular diameter and yolk size.

### 4.4. Heart Rate Detection and Locomotor Activity Assay

To measure heart rate, fish larvae at 72 hpf were anesthetized by soaking in 168 mg/L buffered tricaine methanesulfonate (MS222, Sigma-Aldrich, Merck Life Science, Milan, Italy) and examined under a DMi8 inverted microscope (Leica Microsystems, Milan, Italy). Heartbeats in 60 sec were manually counted from individual video recorded for five randomly selected embryos from each experimental group. Heartbeat counts were carried out twice by two distinct operators.

The locomotor behaviour of control and treated larvae was assessed at 119 hpf. Each 96-well plate containing larvae was transferred into the Zebralab video-tracking platform (ViewPoint Behavior Technology, Lyon, France), and larvae were allowed to acclimate for 15 min with the illumination of the testing chamber set at 50%. The activity levels of larvae were then immediately recorded for 30 min in the same illumination condition, to assess the average swim speed and distance travelled. The video output was analysed with the ZebraLab Tracking Mode v3.22.3.89 (ViewPoint Behavior Technology, Lyon, France), and the raw data was processed with ViewPoint FastData Manager v2.4.0.2510 (ViewPoint Behavior Technology, Lyon, France). The locomotor behaviour analysis was performed three times, at the same time of the day, using independent batches of larvae.

### 4.5. RNA Extraction, Reverse Transcription and Real-Time Quantitative PCR

Total RNA from batches of 50 control and treated embryos at 30 hpf was extracted by using the RNeasy Plus Mini kit (Qiagen, Milan, Italy). Concentration and quality of RNA samples were determined by spectrophotometer readings and agarose gel electrophoresis. Reverse transcription was performed by using the High-Capacity cDNA Reverse Transcription Kit (Applied Biosystems, Life Technologies Italia, ThermoFisher Scientific, Monza, Italy), according to the manufacturer’s recommendations, and 10 ng of the resulting cDNA samples were used as template for qPCR analysis, adapting previously reported methods [64,65,66]. qPCR experiments were performed from two different batches and all reactions were run in triplicate on a StepOnePlus Real-Time PCR System using SYBR-Green detection chemistry and the oligonucleotide primers indicated in Table S1. In each experiment, a no-template control was included for each primer set. ROX was used as a measure of background fluorescence and, at the end of the amplification reactions, a melting curve analysis was run to confirm the homogeneity of all amplicons. The *rpL13a* and the *actb2* mRNAs were used to normalize all data, in order to account for fluctuations among different preparations. Calculations from qPCR raw data were performed using the comparative Ct method (ΔΔCt).

### 4.6. Chromatin Immunoprecipitation (ChIP) Assay

ChIP experiments were performed by adapting previously reported methods [67,68,69,70]. Briefly, zebrafish control and treated embryos were removed from the chorion at 30 hpf, and the yolk was disrupted mechanically by incubation in deyolking buffer (55 mM NaCl, 1.8 mM KCl, 1.25 mM NaHCO_3_) [71]. After deyolking, embryos were dispersed into single cells by transferring them to a PBS solution containing 0.25% trypsin and 1 mM EDTA followed by incubation for 20 min at 28.5 °C [72]. Trypsinization was stopped with 1 mM CaCl_2_ and isolated cells were fixed with 1% formaldehyde in PBS for 8 min at room temperature, followed by quenching of the fix with 125 mM glycine.

Cross-linked cells were washed three times with ice-cold PBS, collected by centrifugation and incubated in cell lysis buffer (50 mM Tris HCl pH 8, 10 mM EDTA, 1% SDS, 1 mM PMSF) containing 1:100 protease inhibitor cocktail (Sigma-Aldrich, Merck Life Science, Milan, Italy). Chromatin was sonicated using a Sonorex Digitec DT103H high-power ultrasonic bath (Bandelin, Berlin, Germany) to an average fragment size of 150 to 500 bp, as determined by agarose gel electrophoresis.

Aliquots of chromatin were immuno-cleared with salmon sperm DNA/protein A-sepharose and immunoprecipitated overnight at 4 °C either in the absence of antibodies or with the anti-acetyl histone H4, the anti-trimethyl H3K4me3 or the anti-trimethyl H3K9me3 antisera purchased from Millipore, Merck Life Science, Milan, Italy (cat# 06-866, 07-473 and 07-442, respectively). An equivalent aliquot of chromatin, referred to as input control, was withdrawn and processed as the immunoprecipitated samples. The immune complexes were washed sequentially, eluted with a buffer consisting of 1% SDS and 0.1 M NaHCO_3_, digested with 0.2 μg/μL RNase A at 37 °C for 2 h and treated with 0.2 μg/μL proteinase K in 0.3 M NaCl at 65 °C for 4 h to reverse the cross-links.

DNA from chromatin samples was then extracted with phenol/chloroform, precipitated with ethanol and dissolved in 50 μL of nuclease-free water. Finally, DNA samples were quantified by readings in a Qubit Fluorometer (Invitrogen, Life Technologies Italia, ThermoFisher Scientific, Monza, Italy) using the Quant-iT dsDNA HS assay kit (Invitrogen, Life Technologies Italia, ThermoFisher Scientific, Monza, Italy).

The enrichment of *cat* and *ldha* gene promoter sequences in 100 pg aliquots of ChIPed DNA and input controls was examined by qPCR, as described above, using the oligonucleotide primers indicated in Table S1. Ct values obtained for each IP sample were normalized to Ct values of the Input, and the percent of Input values were calculated separately for each of the three replicates of an IP sample using the following formula 2 × 100^normalized Ct^ and finally averaged.

### 4.7. Determination of Total Antioxidant Capacity and ROS Production

Total antioxidant capacity was determined by using the OxiSelect Total Antioxidant Capacity Assay Kit (Cell Biolabs, CliniSciences, Guidonia Montecelio, Italy), which relies on the principle that the reduction of divalent to monovalent Copper ion can form a stable-coloured complex with a chromogenic reagent. The net absorbance values measured at 490 nm in lysate samples derived from batches of 10 control and treated embryos at 30 hpf were compared with a uric acid standard curve and were proportional to the capacity of antioxidant species in samples. Each sample was assayed in duplicate, and results were expressed as μM Copper reducing equivalents.

ROS level was quantified by staining control and treated embryos in the dark for 15 min with 5 µM of the fluorescent dye 2,7-dichloro-dihydro-fluorescein-diacetate (DCFH-DA) green-fluorescent dye (Merck Life Science, Milan, Italy). After incubation, embryos were washed thrice with E3 medium and anesthetized as described above. The green fluorescence of oxidized DCF was visualized using a Leica M205-FA stereomicroscope and quantified using ImageJ software.

### 4.8. Acridine Orange Staining

Control and treated zebrafish larvae at the desired stage were incubated in E3 medium containing 2.5 µg/mL of the vital dye acridine orange (Sigma-Aldrich, Merck Life Science, Milan, Italy) for 20 min at room temperature in the dark. After rinsing five times in fresh E3 medium to remove the excess dye, the fish were anesthetized as described above and apoptotic foci were identified with a Leica M205-FA fluorescence stereomicroscope.

### 4.9. Caudal Fin Regeneration Assay

Zebrafish embryos at 24 hpf were placed in 96-well culture plates (1 embryo/well) and reared either in the absence or in the presence of CM at 75 µg/mL. At 48 hpf, embryos were dechorionated, anesthetized and the caudal fin primordia were amputated with a surgical razor blade just posterior to the notochord. Fish were then rinsed with fresh E3, individually transferred into new plates and allowed to regenerate either in sterile E3 or CM for 2 days at 28.5 ± 0.5 °C. Further control groups from the same batches of embryos included both normal and CM-treated un-amputated fish that were dechorionated and anesthetized in the same way. For morphological observation of fin regeneration, the larvae were anesthetized and imaged at 0, 24 and 48 hpa using a Leica M205-FA stereomicroscope. The fin area for each larva was quantified with ImageJ and normalized with respect to those of control un-amputated fish at the same stage.

### 4.10. Statistical Analysis

The criteria of normality of data and homogeneity of variance were assessed by using the Kolmogorov–Smirnov test and Levene’s test, respectively. For normally distributed data, one-way ANOVA analysis followed by Tukey HSD test was performed. Otherwise, non-parametric Kruskal–Wallis was used. The differences among test groups were assessed to be significant at *p* < 0.05 (*), *p* < 0.01 (**) and *p* < 0.001 (***).

## 5. Conclusions

In summary, our multiparametric investigation of the biological effects established in CM-treated zebrafish embryos allowed the identification and characterization of antioxidant, pro-survival and pro-regenerative outcomes. To the best of our knowledge, there is no study so far to investigate the effects of CM derived from Wharton’s jelly mesenchymal stem cells in a whole living vertebrate organism from a developmental, molecular and behavioural perspective. We are fairly confident that our evidence will pave the way for future therapeutic applications.

## Data Availability

No new datasets were created or analysed in this study.

## Supplementary Materials

The following supporting information can be downloaded at: https://www.mdpi.com/article/10.3390/ijms241713191/s1.
