# Supplementary material for: Antioxidant, Pro-Survival and Pro-Regenerative Effects of Conditioned Medium from Wharton’s Jelly Mesenchymal Stem Cells on Developing Zebrafish Embryos
Source: Int J Mol Sci. 2023 Aug 25;24(17):13191. doi: 10.3390/ijms241713191 (PMC10488285; doi:10.3390/ijms241713191)
Supplement: Supplementary file 1 [file ijms-24-13191-s001.zip › ijms-2561348-supplementary.pdf]

Table S1. List of gene-specific oligonucleotides used in the RT-qPCR and ChIP-pPCR experiments.

Target gene	Forward (F) and Reverse (R) sequences (5' to 3')	Length	Amplicon size (bp)	Figures in which oligonucleotide pair was used
baxa	F: GACAGGGATGCTGAAGTGAC<br>R: GTCGGCTGAAGATTAGAGTTG	20<br>21	158	6A
bcl2l1	F: TAGACAACCATATTCAACCCTG<br>R: ACCCGTGAGCAAGGTCATTC	22<br>20	149	6A
bdnf	F: TTGGCGAAGAGCGGACGAAT<br>R: ATAGTAACGAACAGGATGGTCA	20<br>22	154	4A
bim	F: CAGCGATTCTGTGCCAGGTT<br>R: AGTTCACGAGCGACCACCAT	20<br>20	159	6A
brg1	F: CTTCACTGGAGGCATCGTTC<br>R: AAGAGCAGCACTTTGTGGTTG	20<br>21	114	4A
casp3a	F: TACAATGACCAGACAGTTGCG<br>R: ATCCACAGATGTATCAGTGCC	21<br>21	144	6A
casp8	F: AGGTTCAGGAATCAGAGTCAC<br>R: ATTGTGCCAGCCGAAGAGTTT	21<br>21	131	6A
cat	F: ATGAAGCCGAGAGAGAGCGT<br>R: TCAGCGTTGTGTTTATCCAGG	20<br>21	154	4A
foxO3a	F: CAGATTTATGATTGGATGGTCC<br>R: ACTATGGAGCGACAGGTTATG	22<br>21	111	4A
ldha	F: GTTGGAATGGTTGGAATGGCT<br>R: CTTGTGCGTCTTGAGAAACAG	21<br>21	147	4A
mcl1a	F: TACCGACGATGAAGCGAGTG<br>R: CTGATGAAGTCCAGAGAGGC	20<br>20	115	6A
nrf2	F: TGCTGTCACTCCCAGAGTTG<br>R: TGTTTGAGCCGAGCCGAGAT	20<br>20	152	4A
sirt1	F: CTGTAAGCATAAGGTGGACTG<br>R: TACGGGACATCAGACGGACA	21<br>20	93	4A
sirt6	F: GACACGGTGGTCGGAGTGA<br>R: TCGCCTGCTCGCCTCATCA	19<br>19	168	4A
sod2	F: TGTGCTAACCAAGACCCTTTG<br>R: AACGCTCGCTGACATTCTCC	21<br>20	160	4A
actb2§	F: ATCACACCTTCTACAACGAGC<br>R: GGCATACAGGGACAGCACAG	21<br>20	173	4A and 6A
rpL13a§	F: AGGTGTGAGGGTATCAACATC<br>R: TTGGTTTTGTGTGGAAGCATAC	21<br>22	170	4A and 6A
p-cat	F: ACATCTATGAACCAAACCATTGA<br>R: ATGACATCTTTCCCAGTTACAG	23<br>22	230	4B
p-ldha	F: GTAGAGCAAGTAGTTTGACCG<br>R: TGTGGCATAGGCAGTATTGAC	21<br>21	147	4B

§ The *actin-beta2* (*actb2*) and *ribosomal protein L13a* (*rpl13a*) mRNAs were used to normalize all RT-qPCR data.
